# Amodiaquine failure associated with erythrocytic glutathione in *Plasmodium falciparum *malaria

**DOI:** 10.1186/1475-2875-6-47

**Published:** 2007-04-23

**Authors:** Lina Zuluaga, Adriana Pabón, Carlos López, Aleida Ochoa, Silvia Blair

**Affiliations:** 1 Grupo Malaria, Universidad de Antioquia, Calle 62 # 52–59, Torre 1, Piso 6. Laboratorio 610, Sede de Investigación Universitaria (SIU), Universidad de Antioquia, Medellín, Colombia; 2 Facultad de Ciencias Exactas y Naturales, Universidad de Antioquia, Medellín, Colombia

## Abstract

**Objective:**

To establish the relationship between production of glutathione and the therapeutic response to amodiaquine (AQ) monotherapy in *Plasmodium falciparum *non-complicated malaria patients.

**Methodology:**

Therapeutic response to AQ was evaluated in 32 patients with falciparum malaria in two townships of Antioquia, Colombia, and followed-up for 28 days. For every patient, total glutathione and enzymatic activity (glutathione reductase, GR, and γ-glutamylcysteine synthetase, γ-GCS) were determined in parasitized erythrocytes, non-infected erythrocytes and free parasites, on the starting day (day zero, before ingestion of AQ) and on the day of failure (in case of occurrence).

**Results:**

There was found an AQ failure of 31.25%. Independent of the therapeutic response, on the starting day and on the day of failure, lower total glutathione concentration and higher GR activities in parasitized erythrocytes were found, compared with non-infected erythrocytes (*p *< 0.003). In addition, only on the day of failure, γ-GCS activity of parasitized erythrocytes was higher, compared with that of healthy erythrocytes (*p *= 0.01). Parasitized and non-parasitized erythrocytes in therapeutic failure patients (TF) had higher total glutathione on the starting day compared with those of adequate clinical response (ACR) (*p *< 0.02). Parasitized erythrocytes of TF patients showed lower total glutathione on the failure day, compared with starting day (*p *= 0.017). No differences was seen in the GR and γ-GCS activities by compartment, neither between the two therapeutic response groups nor between the two treatment days.

**Conclusion:**

This study is a first approach to explaining *P. falciparum *therapeutic failure in humans through differences in glutathione metabolism in TF and ACR patients. These results suggest a role for glutathione in the therapeutic failure to antimalarials.

## Background

Malaria is a parasitic disease, which has the highest morbidity/mortality rate in tropical countries [1]. The causal agent with the highest lethality rate for this disease and resistance to antimalarials in the world is *Plasmodium falciparum *[2]. In Colombia, there have been reports about resistance of *P. falciparum *to antimalarial drugs since 1960. In Antioquia, Colombia, 97% and 30% of therapeutic failure to the 4-aminoquinolines, chloroquine (CQ) and amodiaquine (AQ) respectively, have been reported [3,4]. Therefore, CQ is no longer used in Antioquia and the use of AQ in combination with sulphadoxine-pyrimethamine (SP) was suggested since 1985 and until 2006 [4].

Haemoglobin is degraded by *Plasmodium *during intraerythrocytic stages in a process where haem is released and converts itself into a toxic molecule, because this parasite lacks haem oxygenase and produces reactive oxygen species (ROS) [5]. The parasite detoxifies haem simultaneously by polymerization and degradation. In the first case, the parasite converts almost 30% of haem in haemozoin or malarial pigment. Haem toxicity is avoided too, through cytosolic degradation by reduced glutathione (GSH). This can degrade haem, whether it is free in solution or it is bound nonspecifically to protein, when dissolved in erythrocyte membranes or loaded into intact erythrocytes [6,7]*Plasmodium *is endowed with a machinery to detoxify ROS. This antioxidant system comprises GSH and thioredoxin redox system, superoxide dismutase, NADPH and a vigorous pentose phosphate pathway, but it lacks catalase and glutathione peroxidase [8-10].

Glutahione is synthesized by the step-limiting enzyme γ-glutamyl cysteine synthetase (γ-GCS) and by glutathione synthetase (GS). This thiol found in its reduced state inside all cells, contributes to maintaining intracellular redox state; besides, it has been suggested that GSH is involved in drug resistance both as a cofactor for enzymatic reactions and by helping to mediate resistance as a source of reductive detoxification of haem. After its reaction with a free radical, GSH changes its oxidation state (GSSG). Glutathione reductase (GR) NADPH is the enzyme responsible for keeping glutathione in its reduced state [9,10].

The resistance of *P. falciparum *to the 4-aminoquinolines, such as CQ, has been shown to be associated with modification of drug transport by the membrane proteins P-glycoprotein homologue 1(Pgh1) and the *P. falciparum *chloroquine resistance transporter (pfcrt) [10]. An additional mode of resistance has been suggested that is connected to an increase of GSH levels, which compete with the drug for degrading the haem toxin [7].

Meierjohann *et al. *[11] showed that growth inhibition of *P. falciparum *CQ-sensitive strain (3D7) by L-buthionine-(S, R)-sulfoximine (BSO), a specific inhibitor of γ-GCS and by methylene blue (MB), an inhibitor of the GR, was significantly more pronounced than inhibition of *P. falciparum *CQ-resistant strain (Dd2) growth by these drugs. These results correlate with the higher levels of total glutathione in *P. falciparum *Dd2. In addition, they suggest that maintenance of intracellular GSH in *P. falciparum *Dd2 is mainly dependent on GSH synthesis, whereas in *P. falciparum *3D7 is regulated via GR [11].

Studies *in vivo *on *Plasmodium berghei*-infected mice confirm that drugs which alter GSH intracellular concentrations, change *Plasmodium *susceptibility to the CQ [12]. The correlation between GSH level and resistance CQ is supported by the higher expression of γ-GCS [13]. It has been shown that acquisition of CQ resistance in *P. berghei *is associated with a significant increase in parasite glucose 6-phosphate deshydrogenase (G6PD) activity and GSH. Combination of CQ with an inhibitor of G6PD or BSO significantly increased sensitivity of resistant parasites to CQ and increased the survival period of the infected mice [14]. Other studies *in vivo *on *P. berghei *and *Plasmodium vinckei-*infected mice showed that drugs, such as acetaminophen, indomethacin and disulphiram, which produce an indirect decrease in GSH, potentiate the antimalarial action of CQ and AQ sub-curative doses [15].

This study examined whether there exists a relationship between the total glutathione level in patients with malaria by *P. falciparum *and therapeutic failure to AQ, since CQ has not actually been used as a monotherapy. Possible variations of total glutathione levels and GR and γ-GCS activities were determined in function of the therapeutic response in parasitized erythrocytes, non-infected erythrocytes and free parasites. It should help to understand the phenomena of therapeutic failure to the 4-aminoquinolines.

## Materials and methods

Within the framework of the evaluation of the resistance to antimalarics by RAVREDA (Red Amazóniza de Vigilancia a la Resistencia de Drogas Antimaláricas), during 2003 and 2004, clinical and parasitological therapeutic response to monotherapy with AQ was assessed in patients with non-complicated *P. falciparum *malaria, according to the WHO Protocol 2000 that classifies the response in adequate clinical response (ACR) and therapeutic failure (TF). Patients were from Turbo and El Bagre, two townships in Antioquia, Colombia. In this study, the therapeutic failure was higher than permitted (>26%) by National Health Ministry treatment guidelines in Colombia and, therefore, the study was carried out only with 32 patients. Initial malaria diagnosis was carried out with thick and thin smear; these were Field and Giemsa stained respectively. Parasitaemia was calculated by counting the number of asexual forms by 200 leucocytes [16]. Samples of venous blood with anticoagulant CPD were taken on the starting day (zero day, before ingestion of AQ) and failure day for those patients who failed (before the rescue treatment). Samples were kept in liquid nitrogen until their processing. Total glutathione concentration and the enzymatic activity of GR and γ-GCS were determined in infected erythrocytes, non-parasitized erythrocytes and free parasites.

### Ethical considerations

A written informed consent from each patient was obtained and signed before being included in the research project. This study was approved by the Ethics Committee of the Facultad de Medicina of the Universidad de Antioquia.

### Sample thawing

Blood samples were thawed according to the workshop protocol suggested in the course "Molecular Approaches to Malaria" – International Centre for Engineering and Biotechonology [17].

### Separation of parasitized and non-parasitized erythrocytes by Percoll gradients

In thawed samples, parasitized and non-parasitized red blood cells were separated by Percoll gradients according to the methodology described by Omodeo-Sale *et al *2003 with some modifications [18]. The thawed red blood cells pellet was taken to a haematocrit of 20 – 25% with serum-free RPMI and fractionated onto a Percoll/4% sorbitol (wt/vol) gradient: (from bottom to top) 90, 80, 70, 60 and 40% percoll. Resuspended erythrocytes were over-layered on the gradient and centrifuged 2000 RPM at room temperature (RT) for 20 min. Three layers were obtained: an upper layer which corresponds to parasitized red blood cells; an intermediate layer which contained a mix of parasitized and non-infected erythrocytes, and a bottom layer corresponding to non-parasitized erythrocytes. The upper layer was removed. To enrich the non-parasitized erythrocyte fraction, the other two lower layers were mixed and fractionated again in the same Percoll-sorbitol gradient. All layers were washed twice with fresh serum-free RPMI. 5 μl from the upper layer and 2.5 μl of the bottom layer were suspended in 200 μl of distilled water.

### Separation of free parasites from parasitized red blood cells infected with *P. falciparum*

The free parasites were obtained according to Hsiao protocol with some modifications [19]: the pellet of parasitized red blood cells obtained by separation with Percoll gradients was taken and the same volume of 0.1% saponin was added; it was mixed with vortex and then incubated during 10 minutes at 37°C, by mixing gently. It was centrifuged at 2,000 rpm for 15 minutes at RT. The pellet obtained had the free parasites which were washed with buffer phosphate by centrifugation at 10,000 rpm during 5 minutes at RT until the red color disappeared. The pellet of free parasites was diluted in 200 μl of distilled water.

### Enzymatic activity and quantification of total glutathione

The enzymatic activity of glutathione reductase (GR) and γ-glutamylcysteine synthetase (γ-GCS), protein concentration and total glutathione content were determined in parasitized and non-parasitized erythrocytes and in free parasites, and were carried out in duplicate.

#### Determination of glutathione reductase activity

This was carried out according to the method described by Calberg I and Mannervik B 1985 [20]. Glutathione reductase enzymatic activity is based on the decay of the absorbance of NADPH at 340 nm measured during a 5-minute period at 30°C.

#### Determination of γ-glutamyl cysteine synthetase activity

This was determined according to the method described by Estrada del Cueto et al. 1999 [21]. This technique assumes that enzymatic activity is equal to the change in NADH absorbance at 340 nm during 60 minutes at 37°C. The absorbance obtained while adding pyruvate/kinase enzymatic mixture (PK/LDH Sigma-Aldrich) was taken as zero time; it was incubated and absorbance was read again.

#### Determination of total glutathione concentration by HPLC

The concentration of total glutathione (oxidized and reduced) was analysed as mBBr derivatives (thiolyte^® ^Calbiochem) by reversed-phase HPLC. The method suggested by Luersen *et al. *2000 was followed with some modifications [22]: to 20 μl of sample (parasitized erythrocytes, non-infected erythrocytes or free parasites) in 1.5-ml Eppendorf tubes placed on ice and protected from light were added 10 μl of NABH_4_, in 0.066 M NaOH and 33% (v/v) dimethyl sulphoxide (DMSO), 6 μl of 2 mM EDTA and 1.65 mM dithiothreitol, 6 μl of octanol and 14 μl of 1.8 M HCl. After three min 70 μl of 1 M ethylmorpholine buffer (pH 8.5), 134 μl of deionized water and 14 μl of 5 mM thiolyte^® ^were added. The derivatization was carried out at 70°C for 10 min in the dark and was terminated by adding 26 μl of 100% acetic acid. After 20 min on ice in the dark, the sample was extracted with 200 μl of dichloromethane and centrifuged at 10,000 rpm at RT for two minutes. The supernatant, which is the water-soluble phase, was taken and filtered through a 0.45-μm nylon membrane. The filtered samples were stored at -20°C, protected from light until their injection in the HPLC. 20 μl obtained from the water-soluble phase were injected on to a LiChroCART^® ^100 RP-18 (5.0 μm) reverse phase HPLC. The column was eluted at a flow rate of 0.55 ml/min by the following gradient of solvent A (0.25% acetic acid) and solvent B (100% acetonitrile): 0 min, 100% solvent A; 5 min, 90% solvent A; 20 min, 85% solvent A, 25 min, 0% solvent A. The effluent was monitored by a fluorescence spectrophotometer (excitation 400 nm; emission 475 nm). Under these conditions, the glutathione-thiolyte^® ^adduct had a retention time of 13.9 min and cysteine-thiolyte^® ^of 10.07 min. Cysteine (Cys) and GSH were used as external standards with recovery rate of 123.8 ± 6.6% and 74.8 ± 8.2% respectively. Two calibration curves were elaborated due to concentration variation of glutathione in the samples and in doing the regression analysis (lineal model). The obtained sensibility was 3.2 pmol, which is found in the quantity range detected when working derivatized compounds with monobromobimane [23].

#### Determination of protein concentration

Protein determination to relate enzymatic activity and the glutathione content was carried out by the method of Lowry *et al *1951 [24].

### Statistical analysis

The data were analysed by the one-way variance analysis (Mann-Whitney test) in order to compare mean of total glutathione concentration and GR and γ-GCS activities in parasitized red blood cells, non-parasitized and free parasites in function of the therapeutic response (ACR and TF). A matching test was applied (Wilcoxon test) to compare the means in both days (starting and failure day) in the group of patients that had therapeutic failure. A correlation analysis and a simple linear regression were made to assess the association between GR and γ-GCS activity in ACR and FT patients with parasitaemia, and glutathione concentration in all cellular compartments. A confidence level of 95% (α = 0.05 o *p *≤ 0.05) was applied to all estimates and statistical tests. The program SpSS 14 was used for the statistical analysis.

## Results

The therapeutic response to monotherapy with AQ was assessed during 28 days in 32 patients with non-complicated malaria by *P. falciparum*; 21 patients came from the township of Turbo, and 11 from El Bagre. Mean age was 27 years (8 – 60) old and 17 of them were male. The 32 patients evaluated were divided into two groups according to therapeutic response to AQ; 22 of them had ACR and 10 TF (31.25%). On the starting day (zero day), mean parasitaemia was 8,699 (880 – 31,960) rings/μl for ACR patients and 9,944 (580 – 38,400) rings/μl for TF patients; on the failure day, mean parasitaemia was 1,681 (26 – 8,640) parasites/μl.

### Glutathione concentration and enzymatic activity in malaria patients before treatment (starting day)

On the starting day for all patients, total glutathione and GR and γ-GCS activities between parasitized and non-parasitized erythrocytes were compared. Non-parasitized erythrocytes had higher total glutathione than the infected ones, both in ACR as well as FT patients (ACR = 16.324 ± 4.992 nmol/mg protein vs 5.612 ± 2.119 nmol/mg proteins, *p *< 0.001 and TF = 21.636 ± 4.149 nmol/mg protein vs 11.725 ± 4.833 nmol/mg proteins, *p *= 0.003). Also for both therapeutic response groups was found that GR activity was statistically lower in the healthy erythrocytes compared to parasitized erythrocytes (ACR = 23.691 ± 8.145 UI/mg protein vs 34.927 ± 14.777 UI/mg proteins, *p *= 0.015 and TF = 18.594 ± 5.442 UI/mg protein vs 30.103 ± 10.171 UI/mg proteins, *p *= 0.021). Differences were not found in the γ-GCS activity between both compartments for any of two groups of therapeutic response (*p *> 0.05) (Table 1).

**Table 1:** 

	**Parasitized Erythrocytes**			**Non-parasitized erythrocytes**			Free parasites*		
	
	**ACR Starting day**	**Failure**		**ACR Starting day**	**Failure**		**ACR Starting day**	**Failure**	
						
		**Starting day**	**Failure day**		**Starting day**	**Failure day**		**Starting day**	**Failure day**
**GSH (nmol/mg protein)**	5.612 ± 2.119^a, h^	11.725 ± 4.833^b, h, j^	5.847 ± 2.582^c, j^	16.324 ± 4.992^a, i^	21.636 ± 4.149^b, i^	19.093 ± 8.288^c^	2.416 ± 0.073	2.400 ± 0	2.400 ± 0
**GR (mU/mg protein)**	34.927 ± 14.777^d^	30.103 ± 10.171^e^	41.901 ± 17.110^f^	23.691 ± 8.145^d^	18.594 ± 5.442^e^	21.999 ± 8.903^f^	18.700 ± 5.867	19.711 ± 9.337	33.562 ± 14.436
**γ-GCS (mU/mg protein)**	20.692 ± 9.208	17.304 ± 8.478	28.141 ± 14.965^g^	17.444 ± 5.785	13.352 ± 6.793	13.150 ± 7.068^g^	10.043 ± 1.549	9.850 ± 1.501	10.5 ± 2.908

*In free parasites GR and γ-GCS enzymatic activity was not established because the protein quantity found was very low and in some cases not detectable, therefore the values shown correspond to enzymatic velocity (mU/ml).

Total glutathione concentration and glutathione reductase (GR) and γ-glutamylcysteine synthetase (γ-GCS) activities of parasitized erythrocytes non-infected erythrocytes and free parasite obtained from adequate clinical response patients (ACR) and failure therapeutic patients (FT). The values shown correspond to starting day (before ingestion AQ) and to failure day. We used the Mann-Whitney test in order to compare total glutathione and GR and γ-GCS activities in parasitized erythrocytes and non-parasitized erythrocytes on both days, and therapeutic responses groups. In addition, this test was used for to establish differences between total glutathione and enzymatic activity on starting day in parasitized erythrocytes between ACR patients and FT patients. Similarly for non-infected erythrocytes and free parasites. A matching test was applied (Wilcoxon test) to compare total glutathione and GR and γ-GCS activities in parasitized and non-infected erythrocytes and free parasites, between starting day and failure day in FT patients. *P *values statistically significant are shown.

^a, b, c^Total glutathione in parasitized erythrocytes compared with total glutathione in non-infected erythrocytes for every therapeutic response group: ACR patients *p *(*M-W*) < 0.001; FT patients on starting *p *(*M-W*) = 0.003; and FT patients on failure day *p *(*M-W*) = 0.001.

^d, e, f^GR activity in parasitized erythrocytes compared with GR activity in non-infected erythrocytes for every therapeutic response group: ACR patients *p *(*M-W*) < 0.015; FT patients on starting *p *(*M-W*) = 0.021; and FT patients on failure day *p *(*M-W*) = 0.013.

^g^γ-GCS activity in parasitized erythrocytes compared with γ-GCS activity in non-infected erythrocytes for every therapeutic response group: FT patients on failure day *p *(*M-W*) = 0.012.

^h^Total glutathione in parasitized erythrocytes of ACR patients compared with FT patients on starting day *p *(*M-W*) = 0.001.

^i^Total glutathione in non-parasitized erythrocytes of ACR patients compared with FT patients on starting day *p *(*M-W*) = 0.015

^j^Total glutathione in parasitized erythrocytes of FT patients on starting day compared with FT patients on failure day *p *(*Wilcoxon*) = 0.017

When the analysis was done based on the therapeutic response, we found that TF patients have higher total glutathione in parasitized erythrocytes and in non-parasitized erythrocytes compared with those that had ACR (*p *< 0.016). There were no differences for GR and γ-GCS activities for either therapeutic response group (Table 1).

The glutathione content in free parasites was the same in all patients, sometimes it was below the method detection limit (3.2 pmol). Differences in GR and γ-GCS activities were not found for either therapeutic response group.

In correlation analysis and a simple linear regression, TF patients presented a 58% correlation between parasitaemia and GR activity of free parasites, and statistically significant correlations were not found in any of the cellular compartments for ACR patients (always r^2 ^< 0.5, data non-shown).

### Glutathione and enzymatic activity in FT patients

Total glutathione and the activity of both enzymes were studied in the 10 TF patients. On the failure day, it was found that infected erythrocytes had lower total glutathione and higher GR y γ-GCS activities compared with non-parasitized erythrocytes (*p *< 0.014) (Table 1). In the analysis of TF patients based on the day (starting and failure day), was seen a significant decrease in glutathione concentration of parasitized erythrocytes on failure day (11.725 ± 4.833 vs 5.847 ± 2.582, *p *= 0.017) and there was no important changes in total glutathione of non-parasitized erythrocytes or in the GR and γ-GCS activity (Table 1). However, there was 93% correlation between GR activity of parasitized erythrocytes and parasitaemia on the failure day.

## Discussion

In this study, AQ failure in patients with malaria by *P. falciparum *was 31.25%, which is above the established value to stop a treatment schaeme. This is the reason why it was not possible to include a larger number of patients.

Luersen *et al *reported that non-parasitized erythrocytes from ring-synchronous cultures of *P. falciparum *have 44% more GSH than parasitized erythrocytes [22]. Although samples in this study come from malaria patients, this is in agreement with the results, where parasitized erythrocytes had lowered their total glutathione by more than 50%. In non-infected erythrocytes, there is no *Plasmodium*-induced intra erythrocytic oxidative stress and glutathione efflux and oxidation. However, it is important to know if glutathione concentrations in this study correspond to its reduced state, in order to confirm the hypothesis formulated. The higher GR activity that was found on the starting day in the *Plasmodium*-infected erythrocytes could indicate that the contribution of the parasite to glutathione metabolism depends on its reduction rather than on its synthesis. GR activity found in free parasites supports this hypothesis. The 58% correlation between parasitaemia and GR activity in the free parasites in TF patients on starting day should be noted. This might explain a difference in glutathione metabolism not influenced by the presence of the drug and possibly favouring therapeutic failure.

Glutathione could compete with AQ for the haem group, possibly explaining therapeutic failure [7]. When glutathione content and the activity of both enzymes (GR y γ-GCS) in the different compartments studied are compared with therapeutic response on the starting day (before ingestion of AQ), we observed that parasitized and non-parasitized erythrocytes of FT patients had higher amount of total glutathione compared with ACR erythrocytes. This is in agreement with reports by Safeukui *et al.*, who found that trophozoites of *P. berghei *CQ resistant strains isolated from infected and untreated mice have higher glutathione concentration in reduced form than in oxidized form, compared with trophozoites of *P. berghei *CQ sensitive and untreated [14]. The differences in parasitaemia do not explain the results we obtained because, on the starting day, parasitaemia is similar in both groups. A possible explication could be that the resistant parasites have a better redox system and, therefore, may have higher levels of total glutathione, resulting from the *novo *synthesis and reduction of oxidized glutathione. Differences in the enzymatic activity could not be observed in this study because, in the techniques used, NADH or NADPH could be consumed by other enzymes.

The detection limit of total glutathione was 3.2 pmol, which agrees with the report of Ivanov *et al. *that point out a detection limit of 2–5 pmol [23]. Nevertheless, the small amount of parasites possibly caused the total glutathione to be below the detection limit in free parasites and, therefore, could have been the cause for not finding differences.

Also, on failure day it was examined if there was a difference between the parasitized and non-parasitized erythrocytes in the total glutathione and enzymatic activities; a statistically higher total glutathione in non-infected erythrocytes compared with parasitized erythrocytes was found. These results agree with those found on the starting day and therefore could also be explained by the consumption of glutathione, product of the haemoglobin metabolism in the red blood cell infected with *Plasmodium*. In addition, was observed on failure day that GR as well as γ-GCS activities are statistically higher in the infected erythrocytes. It could be assumed that the maintenance of glutathione on the failure day depend on synthesis as well as reduction. This difference between starting and failure day possibly is due to the presence of the drug that increases the number of free radicals and, therefore, the parasite must also increase the glutathione *novo *synthesis in order to compensate the difference in reactive species that were controlled by greater GR activity on the starting day when the drug was not present.

When establishing the comparison between the starting day and failure day in TF patients at AQ, it was observed that, on the failure day, the total glutathione in parasitized erythrocytes was inferior to that of such patients in pretreatment starting day. As was mentioned above, it is possible that the presence of the drug increased the reactive species and thus increased the consumption of glutathione. This does not occur in the non-parasitized erythrocytes on the failure day, where the amount of total glutathione continues to be the same as on starting day. Nevertheless, these differences in the total glutathione of both days could also be attributed to the difference in parasitaemias, since they were lower on the failure day compared with starting day. Changes in enzymatic activities between the starting day and failure day were not observed. However, with the methodology used, was cannot discard that some difference exists.

With the results obtained, was concluded that:

1) Independent of the therapeutic response and of the treatment day (starting or failure), the total glutathione was higher and GR activity was lower in the non-infected erythrocytes compared with parasitized erythrocytes.

2) The higher GR activity in the infected erythrocytes on the failure day could be due to a compensation mechanism by lower total glutathione in this compartment.

3) The patients with TF had higher total glutathione in parasitized and non-parasitized erythrocytes on the starting day compared with the ACR patients, indicating a possible contribution to the therapeutic failure.

4) On failure day, the maintenance of glutathione in parasitized erythrocytes depends on synthesis and reduction, possibly by the increase in the oxidative stress that AQ generates.

5) In the FT patients, the total glutathione found in infected erythrocytes was less on the failure day compared with starting day. This is possibly due to the larger amount of glutathione which is consumed in presence of the drug and also to lower parasitaemia.

This study is the first attempt to explain *P. falciparum *therapeutic failure through differences found in glutathione metabolism in FT and ACR patients. Our results may involve glutathione in the therapeutic response to antimalarials; nevertheless, further studies are needed to prove this relationship. It is also important to compare these results with samples of healthy individuals to rule out other possible differences. In addition, it is important to perform more accurate enzymatic assays to discard differences in the enzymatic activities between the treatment days and between the therapeutic response groups. It would be useful to do a follow-up of the glutathione during and after the malaric episode in patients with different therapeutic response to establish if the glutathion (GSH and GSSG) change during the infection and if they return to the same level when the parasite is not present.

## Authors' contributions

LZ designed and managed the study, made the assays of separation cellular and evaluated enzymatic activity, analysed of data and wrote the first draft of the manuscript. AP designed study, analysed of data, contributed in the standardization of the techniques and cowrote the first draft of the manuscript. AO validated the method for quantification of total glutathione and made injection the samples to HPLC. CL coordinated the validated for quantification of total glutathione and analysed data. SB coordinated the study, analysed data and critically reviewed and suggested changes to the manuscript. All authors read and approved the final manuscript.
